# A robust approach for analyzing and mapping hierarchical brain connectome towards laminar-specific neural networks

**DOI:** 10.1162/imag_a_00543

**Published:** 2025-04-22

**Authors:** Wei Zhu, Guangle Zhang, Xiao-Hong Zhu, Wei Chen

**Affiliations:** Center for Magnetic Resonance Research, Department of Radiology, University of Minnesota, Minneapolis, MN, United States

**Keywords:** resting-state fMRI, hierarchical functional connectivity, random matrix theory based PCA, laminar connectome

## Abstract

Probing neuronal activity and functional connectivity at cortical layer and sub-cortical nucleus level provides opportunities for mapping local and remote neural circuits and resting-state networks (RSN) critical for understanding cognition and behaviors. However, conventional resting-state fMRI (rs-fMRI) has been applied predominantly at relatively low spatial resolution and macroscopic level, unable to obtain laminar-specific information and neural circuits across the cortex at mesoscopic level. In addition, it is lack of sophisticated processing pipeline to deal with small laminar structures in rodent brains. To fill this gap, we conducted a high-resolution rs-fMRI study of mouse brain at ultra-high field and developed an fMRI preprocessing pipeline that features in random matrix theory-based principal component analysis to remove thermal noise, non-rigid image registration strategy to improve head motion estimation, one-time image voxel shift correction to minimize multi-interpolation-induced spatial blur, and improve subject-level alignment to facilitate group analysis. By applying this pipeline to the high-resolution mouse rs-fMRI with atlas-based connectivity analysis, we achieved high-quality hierarchical connectomes covering from large brain regions to cortical layers, and between white matter bundle fibers and cortices in mice. We demonstrate the hierarchical connectomes connecting to three representative brain regions: somatosensory areas, hippocampal regions, and lateral forebrain white matter bundles, showing previously undetected networks. The distinct laminar-specific networks evidence that the spontaneous neuronal activity is not uniform across the cortical layers in the resting brain, consistent with the layer-specific neuronal projection patterns that were observed in AAV viral tracer projections. Additionally, we also observed extended functional connections in areas with sparse viral tracer projections. The feasibility of achieving laminar-specific connectomes with distinct RSNs provides opportunities to study neural circuits and brain functions at multiple scales, though achieving high fidelity and specificity in mapping laminar-specific connectomes may require even higher spatial resolution.

## 1 Introduction

Resting-state fMRI (rs-fMRI) has become a common and essential imaging technique for studying spontaneous brain activity and functional connectivity under the rest state since its first demonstration (Biswal et al., 1995;Raichle, 2015), showing promise for translational research on the central nervous system. In contrast to task fMRI (Kwong et al., 1992;Ogawa et al., 1992,^1993^), rs-fMRI investigates the slow spontaneous fluctuation of the blood oxygen level dependent (BOLD) signal and its temporal correlation among different brain regions in the absence of external stimulation or task performance. The temporal correlation of spontaneous BOLD fluctuation is assumed to be closely associated with the neural synchronization, a phenomenon that has been observed at multiple scales within the central nervous system (Buzsaki, 2006). Thus, rs-fMRI serves as an excellent surrogate of intrinsic brain activity and synchrony, from which functional connectivity involving multiple brain regions can be mapped and identified (Grandjean et al., 2020;Gutierrez-Barragan et al., 2019;Liska et al., 2015;Ma et al., 2017;Ma & Zhang, 2018;Raichle, 2011,^2015^).

So far, rs-fMRI has been applied predominantly at relatively low spatial resolution that is challenging to obtain depth-dependent information across cortical layers. However, evidence exists indicating that the spontaneous neuronal activity is not uniform across the cortical layers in the resting brain (Baek et al., 2016;Choi et al., 2023). First, different types of neurons cluster in different depths of the neocortex, forming cortical layers of varying thickness and functions (Kandel et al., 2000). For example, layer IV mainly consists of inner granular cells with small cell bodies and layer VI is the spindle cell layer with large cell bodies. Differences in cellular structure may cause differences in neural metabolism and neural-hemodynamics across the neocortex and its cellular layers. Second, neurons in different layers have preferential projection targets to other layers or brain regions (Harris et al., 2019), highlighting some layer-specific cortical projection patterns (Whitesell et al., 2020). Third, spontaneous spike activity seems to be the strongest in layer IV and VI (Snodderly & Gur, 1995) while the gamma and alpha local field potential activities are segregated between superficial and deep cortical laminae, showing strong synchronizations within the laminar zones but weak at inter-laminae boundaries (Buffalo et al., 2011;Maier et al., 2010). As rs-fMRI BOLD signal is generally assumed to reflect a vascular low-pass filtering of higher frequency neural activity (Honey et al., 2009), its fluctuation is expected to be highly layer dependent when using adequate imaging resolution. In addition, a recent study states that spontaneous BOLD signals are specifically attributable to an infra-slow brain process that could travel from deep to superficial cortical layers between visual and motor cortices, implying that long-distance BOLD signal fluctuation and synchrony reflect a previously unappreciated degree of laminar specificity (Mitra et al., 2018). Therefore, it is critical to investigate the laminar response of rs-fMRI and to which degree it can reflect the underlying neural synchronization.

By applying a newly developed data preprocessing pipeline to the high-resolution (250 µm in-plane) mouse rs-fMRI data followed by atlas-based connectivity analysis, we obtained high-quality resting-state networks (RSNs), for the first time, from large brain regions down to cortical layers and the sub-cortical nucleus, observing previously indistinguishable networks and neural circuits in the mouse brain with a small brain volume. The hierarchical functional connectivity was not only observed in the iso-cortex and hippocampus, but also in white matter bundles, providing evidence in mapping the neural networks connecting cortex and white matter. Finally, we compared the similarities and differences in functional connectome with structural connectome that is revealed by AAV viral projection (Kuan et al., 2015).

## 2 Methods

Thirteen wild type mice (C57BL/6, 7 male/6 female) were scanned under a protocol approved by the University of Minnesota IACUC. All mice were anesthetized with 1.5% isoflurane and switched to dexmedetomidine (Chu et al., 2021) during the study. Rs-fMRI data acquisition started once the mouse respiration rate stabilized at >140 BPM. The MRI experiments were conducted on a 9.4T/31cm animal scanner (Varian/VNMRJ) using a single loop (1.5 cm diameter) transceiver RF surface coil. Single-shot 2D GE-EPI based rs-fMRI data were obtained with repetition time (TR) = 1000 ms, echo time (TE) = 16 ms, matrix size = 96 × 48, field of view (FOV) = 2.4 × 1.2 cm^2^, and nominal RF pulse flip angle (FA) is the Ernst angle = 55°. Detailed animal preparation, MRI acquisition protocols, and rs-fMRI data processing can be found in the Materials and Methods section in the Supporting Information (SI) file.

## 3 Results

### 3.1 Improved fMRI data preprocessing pipeline

The high-resolution rs-fMRI mouse brain images acquired using the echo-planar image (EPI) method at 9.4 Tesla were preprocessed with the proposed fMRI preprocessing pipeline shown inFigure 1. Four preprocessing branches for managing field map data, functional data, anatomical data, and atlas data were integrated and labeled in different colors, respectively. The main features of this pipeline were: 1) applying random matrix theory-based principle component analysis (RMT-PCA) denoising with patch processing to effectively remove thermal noise with minimum spatial blur (Zhu et al., 2022), 2) enhancing motion estimation accuracy by means of both rigid and non-rigid image registration strategy (Pachitariu et al., 2016;Pnevmatikakis & Giovannucci, 2017;Syed & Foroosh, 2024), 3) minimizing the spatial interpolation steps by combining voxel shifts from various sources such as static magnetic field (B_0_) distortion, head motion, resampling, etc. into one step to minimize image blur, and 4) using flexible and robust image registration strategy at subject level built on Advanced Normalization Tools (ANTs, (Avants et al., 2014)). The outputs of these critical processing steps were evaluated in detail inFigure 2. Specifically, field map based EPI distortion correction restored distorted EPI images to be closer to its anatomical references, increasing the Pearson correlation between EPI and anatomical images by 3.6% (Fig. 2Aand2B). Corrected EPI images further eased the registration to anatomical images and brain atlas. Second, the RMT-PCA denoising algorithm enhanced image temporal signal-to-noise ratio (tSNR) by 2.2 times (Fig. 2Eand2F,Fig. S1B) while it only increased full width at half maximum (FWHM) by 41% (Fig. 2Cand2D). The histogram of the noise images after denoising follows a Gaussian distribution (Fig. 2G), indicating the algorithm largely removed thermal noise. Further, the motion estimation includes both rigid (2D + 3D) and non-rigid processing. Compared to 3D motion estimation only, the new motion estimation strategy leads to the highest correlation to anatomical images (Fig. 2Hand2J) and the least residual images (Fig. 2J).Figure 2Kand2Malso show an example of 2D motion (phase encoding dimension), 3D motion (three translations and three rotations), and non-rigid motion, respectively. Note that the unit of non-rigid motion is in voxel and the brain shape change is varying for different slices (Fig. 2MandFig. S7). To quantify the blur effect introduced by image interpolation after each processing step, we estimated the FWHM of the output EPIs (Fig. 2N). The FWHM increases as interpolation operation increases, and the averaged FWHM after the last interpolation (upsampling) reaches at 1.9 voxels. In contrast, combining multiple processing steps into one leads to an FWHM of 1.5 voxels with the smallest variation.Figure 2Oshows the nuisance signals that were filtered out from the EPI time series. We also compared the image registration quality between Allen mouse brain atlas and anatomical images to averaged functional images (Fig. 2PandFig. S2). Although the brain size and shape varied from mouse to mouse, the co-registered atlas deformed to match each individual brain with good quality.

**Fig. 1.**
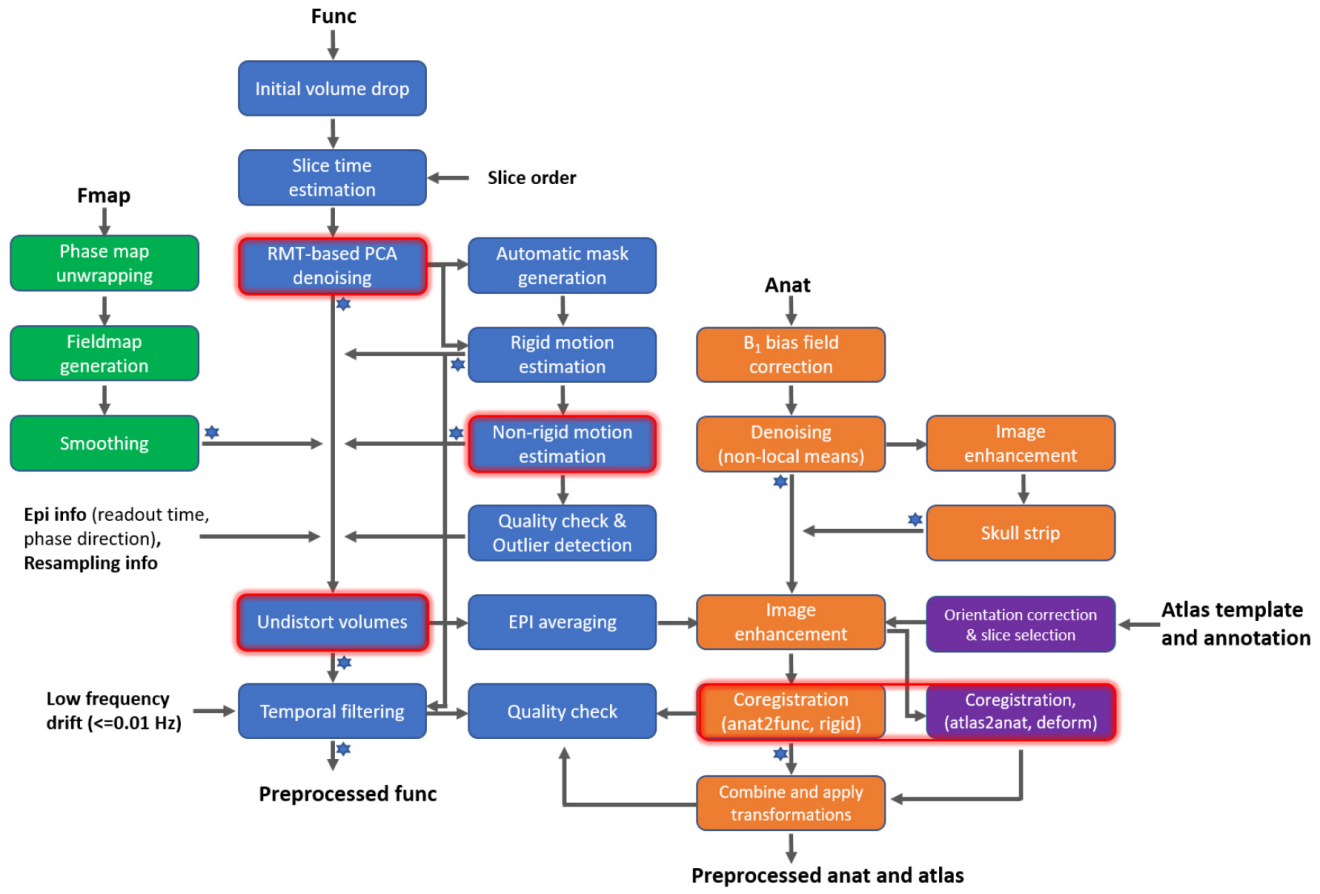
The schematic diagram of the rs-fMRI preprocessing pipeline. Four processing branches are labeled in different colors: green for field map data (Fmap), blue for functional data (Func), orange for anatomical data (Anat), and purple for atlas data. The main Func processing branch features in 1) combining random matrix theory-based principle component analysis (RMT-PCA) denoising to suppress thermal noise without inducing spatial blur (Zhu et al., 2022), 2) enhancing brain motion estimation with non-rigid strategy, and 3) minimizing the spatial interpolations by one-time voxel shift correction from various sources. The main feature of the Anat processing branch for the subject-level alignment was the robust registration strategy built on ANTs (Avants et al., 2008,^2014^). Note that quality check is performed after steps with a star sign.

**Fig. 2.**
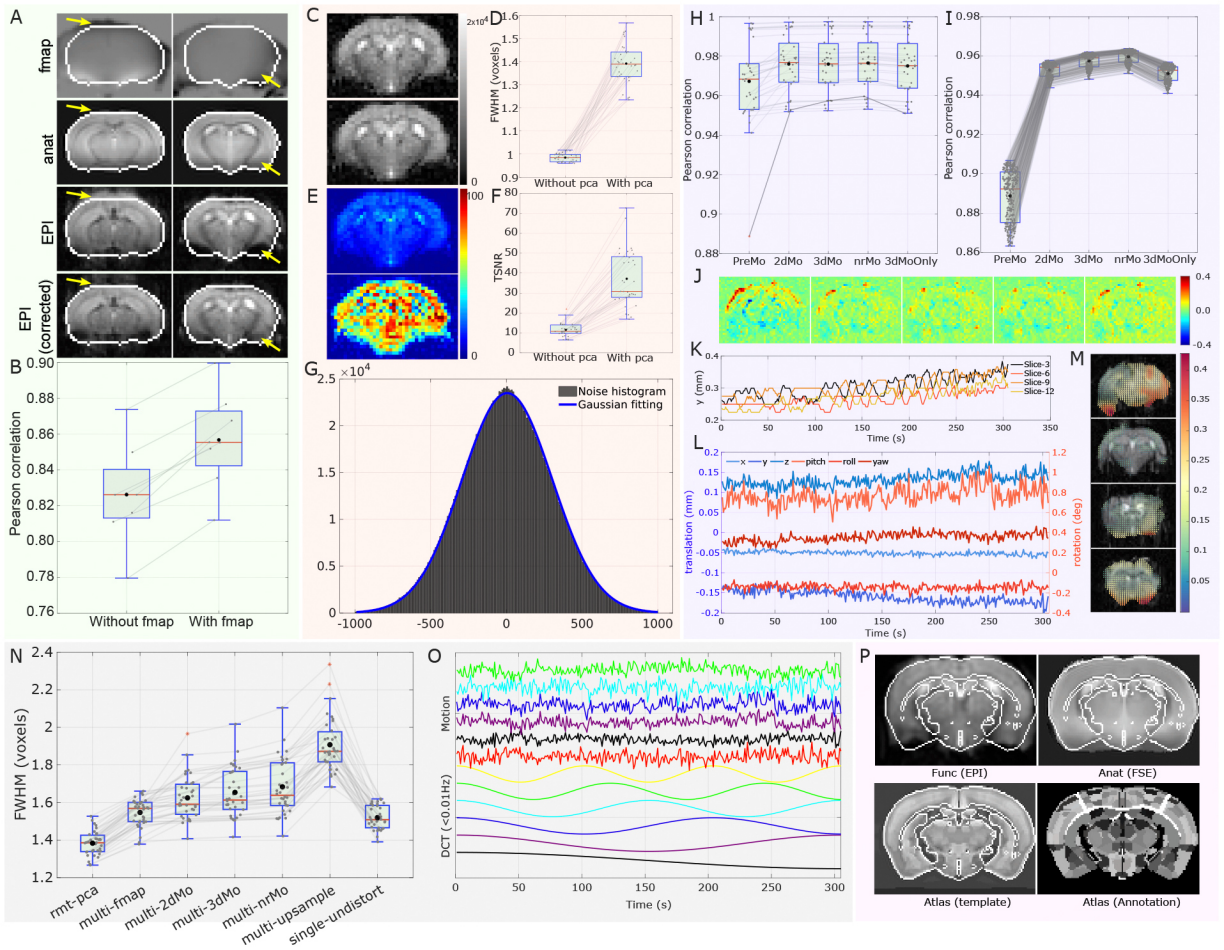
Quality evaluation on specific processing steps: 1) field map based EPI distortion correction (green background): (A) two example slices of field map (fmap), T_2_-weighted anatomical image (anat) as reference, uncorrected EPI images, and field map corrected EPI images; (B) quantification of image restoration by using image correlation between referenced EPI identified for motion correction and anatomical image, 2) RMT-PCA denoising (orange background): (C) Example EPI slice with (top image) and without PCA (bottom image); (D) comparison of FWHM estimated in the brain region with and without PCA; (E) Example tSNR slice with and without PCA; (F) comparison of mean tSNR in the brain region; (G) noise distribution after denoising and Gaussian fitting, 3) motion estimation (purple background): (H) comparison of Pearson correlation between motion corrected EPI with reference EPI for all subjects, and note that the proposed motion correction strategy (from rigid 2D and 3D motion correction to non-rigid correction) leads to the highest correlation, though most subjects have very small motion except for one subject (bolded grey line); (I) further examination of motion correction quality for the particular subject in H; (J) differential image between motion-corrected EPI and reference EPI for each correction step (the image arrangement follows the list along x-axis in H and I); (K) phase encoding pixel shift on example slices after 2D rigid motion correction; (L) translations and rotations after 3D rigid motion correction; (M) pixel displacement map with non-rigid motion correction, 4) blurring in FWHM and temporal regressors (grey background): (N) FWHM estimation for each processing step sequentially (multi-) and FWHM for one single undistortion step (single-); (O) low frequency components and motions that were regressed out from the EPI time series, 5) image registration between EPI, anatomical image, and brain atlas (P on pink background).

**Fig. 3.**
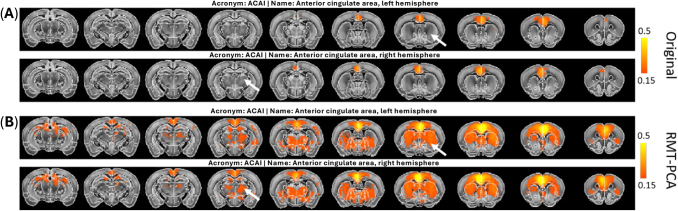
An example of resting-state networks with seeding at anterior cingulate area (ACA) in the left and right hemispheres obtained from (A) original EPI data, and (B) RMT-PCA denoised EPI data. The default mode networks are clearly observed in RMT-PCA denoised data, revealing brain connections between cortices and sub-cortices that are undetectable in original fMRI data (e.g., as pointed by white arrows).

Taking advantage of the segmented Allen mouse atlas (Lein et al., 2007), we performed atlas-based seed connectivity analysis by seeding brain regions of interest at various hierarchical levels (down to cortical layers and thalamic nucleus) on the preprocessed rs-fMRI data to generate hierarchical RSNs. The efficacy of RMT-PCA was further demonstrated on resting-state networks derived from original rs-fMRI data and data denoised by RMT-PCA (Fig. 3). When seeding at anterior cingulate area (ACA), we observed the default mode networks (DMNs) (Grandjean et al., 2020;Gutierrez-Barragan et al., 2019;Liska et al., 2015;Lu et al., 2012;Ma et al., 2017;Ma & Zhang, 2018;Raichle, 2011,^2015^) incorporating ACA, retrosplenial cortex, striatum, and thalamus in RMT-PCA denoised data (Fig. 3B). Instead, at the same correlation strength level, RSNs derived from original data only show limited connection within ACA (Fig. 3A) due to low tSNR. The network connection strength and coverage, and mapping quality were superior by using the RMT-PCA denoised data, that is, when no threshold were given to the seed maps from original data and RMT-PCA denoised data, the average correlation was stronger for RMT-PCA based maps (Fig. S3). Further, RSNs derived from RMT-PCA denoised data reveal convincingly brain connections between cortical and sub-cortical areas that were undetectable in original fMRI data, such as ACA- caudoputamen and ACA- dorsal thalamus (polymodal association cortex related) connections (Fig. 3B), presenting important neural connections between cortical and sub-cortical regions which could belong to DMNs in mouse brains.

### 3.2 Hierarchical resting-state networks in both grey and white matter

**Fig. 4.**
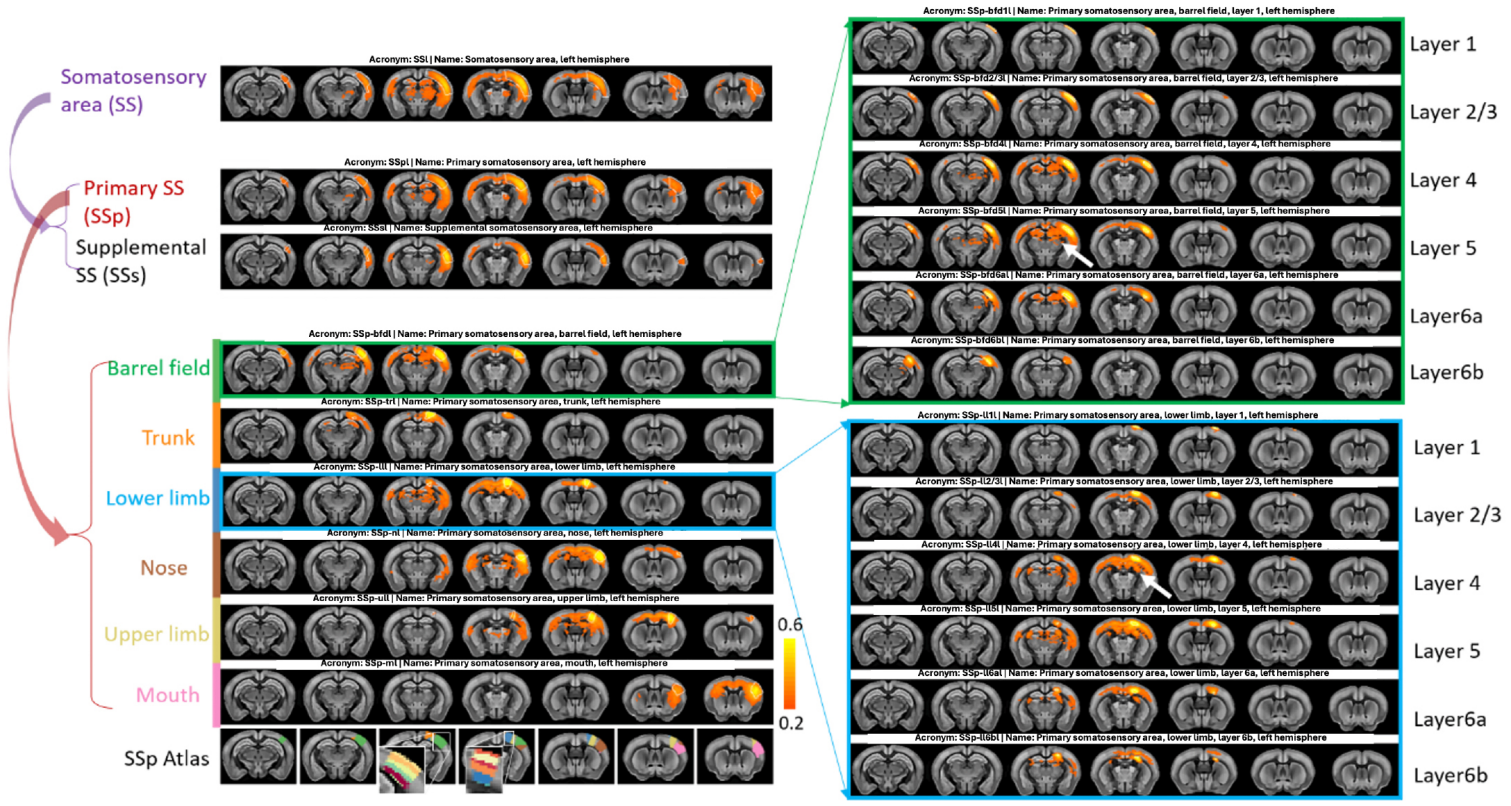
Hierarchical RSNs with seeding in somatosensory (SS) area at different structure levels in left hemisphere based on the RMT-PCA denoised data. High-level RSN (top left panel) is decomposed into sub-RSNs (middle and low left panels) down to the laminar level (right panels) with distinct connection patterns. The decomposed higher-level RSNs are somatosensory area, primary somatosensory area, barrel field, and lower limb, respectively. Similar RSN patterns are observed when seeding the same brain areas in the right hemisphere (Fig. S3). The seeding areas are outlined by white dashed lines. SSp seed atlas is also shown for reference.

Accounting on the largely boosted tSNR (Fig. 2Eand2F) and robust mapping of RSNs brought by the RMT-PCA (Fig. 3), we investigated the feasibility of decomposing regional RSNs to sub-regional RSNs and to laminar RSNs in three selected brain regions of interest: somatosensory area (SS), hippocampal regions, and lateral forebrain white matter bundles.Figure 4shows such hierarchical RSNs with seeding in SS. The brain-wide connections covering sensory and motor area, ACA, striatum, dorsal thalamus, and hippocampus were obvious when averaged rs-fMRI signals in the whole SS area were used as a seeding reference. With seeds set to primary SS and secondary SS, respectively, we observed that main connections from SS were from primary SS, and secondary SS mainly connected with some sensory areas. Interestingly, distinguished RSNs were revealed when sub-regions of primary SS such as barrel field, lower limb, upper limb, mouth, nose, and trunk were seeded (Fig. 4). Strikingly, RSNs at laminar level also demonstrate distinct, robust, and functional connectome patterns. For example, RSNs with seeding at layer 1 of the barrel field have only sparse connections at brain surface area. Layer 2/3 seed maps start to show bilateral and across layer (layer 4) connections. Beside bilateral and across layer connections, layer 4-6a seed maps also show cortical-subcortical connections such as the connections with Ammon’s horn in hippocampal formation and nucleus in thalamus (pointed by a white arrow inFig. 4) with varied connectivity strength (strongest for layers 5). Laminar RSNs from other seeding regions generally show similar trends but with differences. For example, mid-layer seed maps from the lower limb region strikingly show connections with lateral corpus collosum with white matter bundle fibers across two hemispheres (pointed by a white arrow inFig. 4). Interestingly, connections with cerebrospinal fluid (CSF) areas were also observed for layers 4, 5, and 6 in the anesthetized mouse brain, which might be consistent with the coupled electrophysiological, hemodynamic and CSF oscillations as observed in the human brain under the deep sleep state (Fultz et al., 2019). Similar RSNs at different structure levels were also observed when seeding in the right hemisphere (Fig. S4).

Figure S5 is another example showing the hierarchical RSNs with seeding at the hippocampal region (HIP). After decomposing the HIP RSN to sub-region RSNs, we observed a large amount of connectivity similarity shared by the Ammon’s horn RSN and distinguished connectivity for dentate gyrus and indusium griseum. The decomposition of Ammon’s horn (CA) RSN allowed us to identify the different networks for CA 1-3. Further breakdown of CA 1 and CA 3 networks to laminar level reveals local neural circuit connections. For example, CA 1 stratum oriens (so) RSN in green box inFigure S5has weaker connections with thalamus compared to other layers as indicated by the white arrows. CA 3 pyramidal layer (sp) RSN in blue box inFigure S5shows stronger contralateral connections with dentate gyrus as indicated by the white arrows.

As white matter consists of intense bundle fibers and is a bridge for effective communication of ipsilateral and contralateral brain areas as well as cortical and sub-cortical areas, we further explored the RSNs when seeding in lateral forebrain bundle system (lfbs) at various structure levels. The top panel inFigure S6shows the whole-brain connections when lfbs is the seed. Localized and stronger connectivity appears when sub-white matter bundles are seeded (middle panel,Fig. S6). Further breakdown of the corpus callosum seeding generates more localized RSNs involving multiple brain regions shifting from the anterior to the posterior side (low panel,Fig. S6). For example, for the corpus callosum related to body, RSNs show connections with SS-trunk, SS-lower limb, SS-upper limb, SS-nose, ACA, caudoputamen, CA, and some parts of the thalamus across contralateral and ipsilateral hemispheres.

**Fig. 5.**
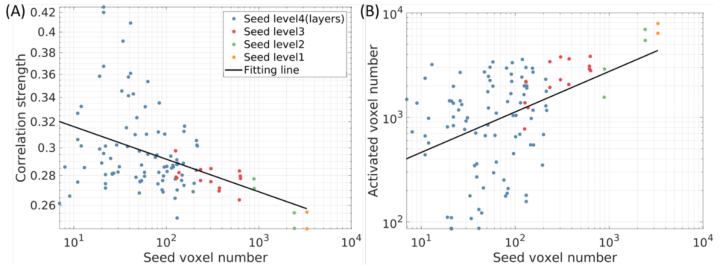
The averaged correlation strength (A) and brain activation area size (B) as a function of seed voxel number for somatosensory area. The smaller the seed size, the stronger the correlation and the smaller the activation area. The lower the structure level (from large brain region to cortical layers), the larger the variation in these measures. Note the axes are in logarithmic scale.

The decomposition of RSNs from a large seed brain region to a smaller brain region indicates the seed size plays a role in averaged correlation strength and correlation area size.Figure 5shows the averaged correlation strength and activated voxel number as a function of seed voxel number when the correlation strength is threshold at 0.2. Generally, correlation strength is inversely proportional to seed voxel number in logarithmic scale whereas the logarithm of activated voxel number is proportional to the logarithm of seed voxel number. However, variation is larger when seed voxel number is small, indicating the improvement of the specificity.

### 3.3 Brain-wide hierarchical correlation matrix and circular graphs

**Fig. 6.**
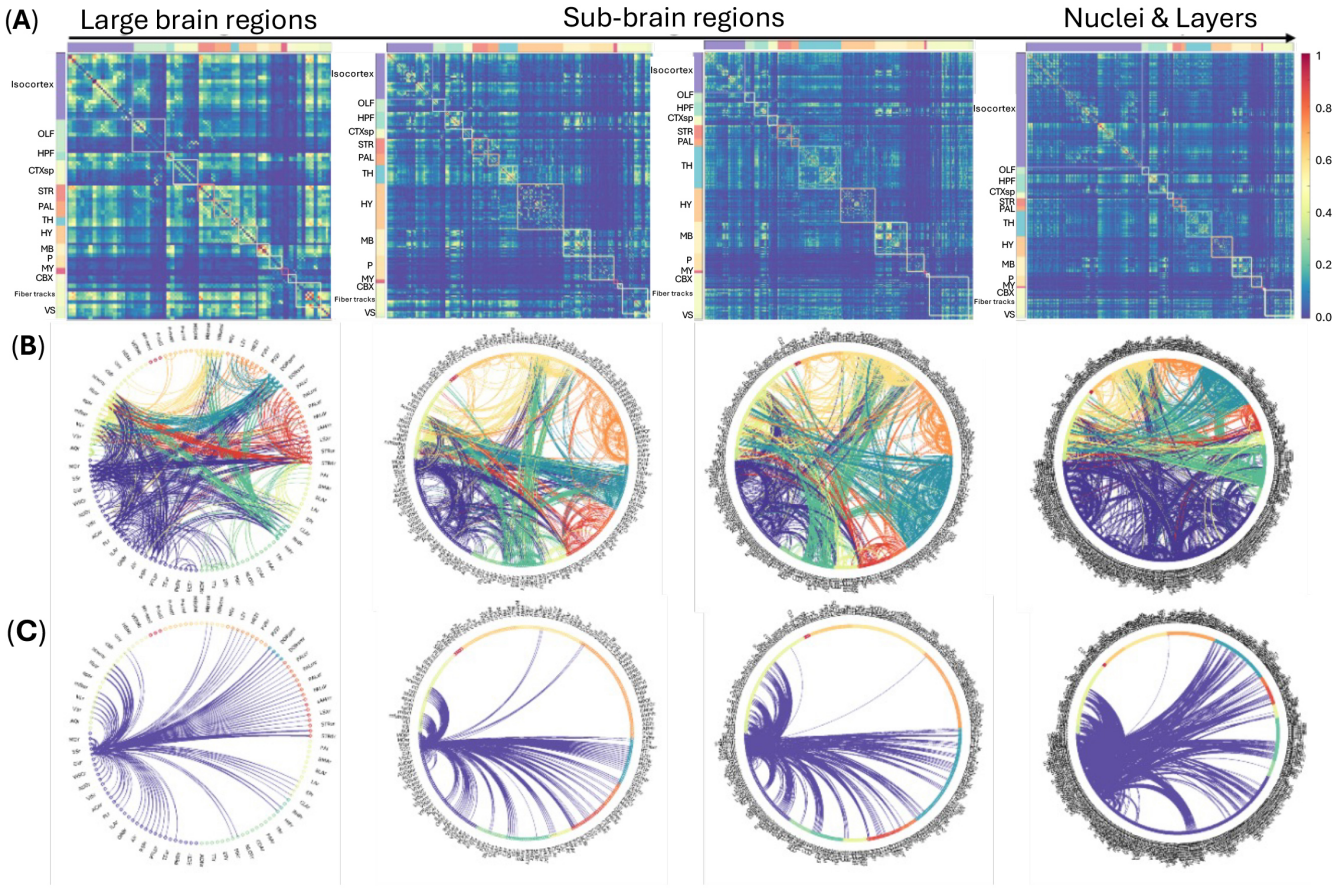
Hierarchical correlation matrices (A) and circular graphs (B) at different structure levels (from large brain region to sub-regions and to nucleus and layer level; left to right column), and circular graphs for somatosensory connectomes at different levels were highlighted in (C). Different colors indicate the top-level brain regions. Abbreviations: OLF, olfactory areas; HPF, hippocampal formation; CTXsp, cortical subplate; STR, striatum; PAL, pallidum; TH, thalamus; HY, hypothalamus; MB, midbrain; P, pons; MY, medulla; CBX, cerebellar cortex; VS, ventricular systems.

By assembling all brain area connections at multiscale into a matrix, we calculated the hierarchical correlation matrices at various structure levels from large brain region to sub-regions, and to nucleus and cortical layers (Fig. 6). As the structure level changes from large brain regions (left column,Fig. 6) to cortical layers (right column,Fig. 6), tremendous details in brain connections are revealed. To visualize differences in functional connectivity with the correlation coefficient strength (Z-score) larger than 0.2, connectograms (or circular graphs) were generated based on the correlation matrices. Connectograms represent the degree of functional connectivity from one specific area of the brain to its various targets for innervation.

### 3.4 Comparison between fMRI RSNs and AAV viral projections

**Fig. 7.**
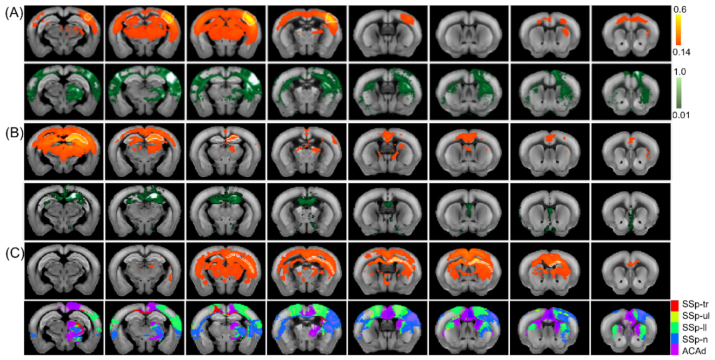
Comparison between rs-fMRI RSNs with seeding at SSp-barrel field (A), CA 1 (B), corpus callosum-body (C), and AAV viral tracer projection targeted in the same corresponding brain areas from Allen Institute. Functional connectome (top rows in each panel) share similarity with Structural connectome (bottom rows in each panel), but with extended connections. Abbreviations: SSp-primary somatosensory area, tr-trunk, ul-upper limb, ll-lower limb, n-nose, ACAd-dorsal anterior cingulate area. The seeding areas for rs-fMRI analysis are outlined by white dashed lines and show high correlation. The bright white areas present the AAV injection location for the AAV viral tracer maps.

To investigate the differences between anatomical projection networks and the observed functional RSNs, we compared three example RSNs with seeding at SSp-barrel field, CA 1, corpus callosum-body with the AAV viral tracer projection with virus injected in the corresponding brain areas (experiment IDs are 127866392 for SSp-bfd, 120436274 for CA 1, and 100141495, 112229814, 112935169, 126908007, 139426984 for combined cortical areas) taken from Allen Institute (Kuan et al., 2015) (Fig. 7). From the AAV viral tracer image, strong structural connections are observed across ipsilateral layers of SSp-barrel field and part of the striatum and thalamus as well as relatively weak connections with contralateral cortical layers and striatum through corpus collosum (Fig. 7A). Sparse viral traces also presented in ACA. Similarly, rs-fMRI SSp-barrel field RSNs also show stronger cortico-cortical and cortico-subcortical connections in ipsilateral brain areas compared to contralateral brain areas. What is different is the extra functional connections to ACA and hippocampus only revealed in rs-fMRI connectivity maps. This extended functional connectome is more obvious when the CA 1 is the seeding or viral injection target (Fig. 7B). As no viral injection experiment was performed in white matter area, multiple primary SS projection data were composited together to compare with the corpus callosum-body RSNs (Fig. 7C). Based on the results, viral projections from multiple brain areas reveal the projection pathways through fibers in corpus callosum. Inversely, when seeding at corpus callosum in rs-fMRI, we observe connectome across those multiple brain regions.

## 4 Discussion

We have demonstrated high-quality brain connectomes from large brain regions decomposed into potential laminar connectivity in resting mouse brains by using a robust data preprocessing pipeline with multiple features tailored for high-resolution fMRI brain mapping. The enhanced tSNR brought by the patch-based RMT-PCA promotes functional mapping fidelity, sensitivity, and specificity (Figs. 2,3&S3). The optimized preprocessing modules minimize spatial blur and augment the alignment accuracy at volume and subject levels (Fig. 2), in favor of the following group-level laminar functional connectivity analysis (Fig. 4,S4-S6). The proposed fMRI preprocessing pipeline provides a valuable tool to analyze spontaneous hemodynamic response, neural circuits, and laminar-specific connectomes. Additionally, it can also work with high-resolution task fMRI mapping on human and animal brains with minimal modified initialization. Although we presented herein three seeding examples of interest and the associated RSNs, the statistical analysis and processing can be performed to identify and investigate other RSNs using the same pipeline and rs-fMRI datasets.

### 4.1 The advantages of high-resolution fMRI

The benefits of high-resolution fMRI include: 1) better structure delineation due to denser spatial sampling, 2) better disentanglement of neural dynamics in local brain regions. As the thickness for each layer in the cortex varies within the region and from region to region, therefore, higher resolution means a better chance to differentiate each layer and their functional dynamics. To demonstrate the advantage of using high-resolution, we compare the seed maps from our data (0.25 mm in-plane resolution) to the same data but downsampled at the spatial resolution of 0.5 mm (Fig. S8). The results clearly show that less specific information can be extracted from different layers in the coarse resolution case. One limitation of this work is that the in-plane resolution of 250 x 250 μm² as applied in this study may be still inadequate for differentiating all six cortical layers with varied thicknesses and some of them could be narrower than 250 μm. For example, the thickness for layers of barrel field in somatosensory area is L1 (0.12 mm), L2-3 (0.25 mm), L4 (0.20 mm), L5 (0.27 mm), L6 (0.32 mm), and the total thickness is 1.16 mm. Higher resolution is better for mapping laminar RSNs; however, we need to make compromises on brain coverage, temporal resolution, fMRI SNR, and tSNR. In the long run, as stronger gradients, higher magnetic fields, and low thermal noise coils are developed, fMRI with even higher spatial resolution (such as 0.1 mm isotropic) is possible, leading to more accurate laminar networks.

### 4.2 Comparing with other fMRI preprocessing strategies

The advent of high-resolution human fMRI (<1 mm) and animal fMRI (<0.3 mm) poses new challenges and requirements on fMRI data preprocessing, leading to a re-evaluation and re-design on the current fMRI preprocessing procedures (Bause et al., 2020;Polimeni et al., 2018;J. Wang et al., 2022). One critical issue related to the current preprocessing procedures is the degradation of spatial resolution due to multiple interpolations and spatial blurring (Botvinik-Nezer et al., 2020;Grootoonk et al., 2000;Polimeni et al., 2018;Power et al., 2017). Minimization of this spatial resolution loss is achieved by either combining all spatial transformations into one (Glasser et al., 2013,^2016^), or upsampling the image (Kang et al., 2007;Kay et al., 2019). However, these belong to a more general voxel-shift transformation category, together with geometric distortion caused by B_0_inhomogeneity, motion correction, etc. In this work, all these steps were combined to be one spatial interpolation step, like the procedure as presented previously (Allen et al., 2022).

Another important step is image denoising to boost sensitivity for detecting the small BOLD signal fluctuation and weak temporal correlation associated with small imaging pixel size, poor tSNR, and weak neuronal activation. Among various noise sources that are common in fMRI, unstructured random thermal noises are always present yet least wanted. It is, therefore, desirable to eliminate them before other processing. This is especially true for high-resolution fMRI where the physiological noise decreases, and the random thermal noise tends to dominate (Liu, 2016). As such, PCA serves as a promising technique because of its utility for effective isolation of unstructured random thermal noise from other sources of BOLD signal fluctuation (Thomas et al., 2002). Previously, we have developed an RMT-based PCA denoising algorithm and applied it to task fMRI (Zhu et al., 2022). In this work, we further adopted it to the proposed pipeline as a feature and compared its performance with original rs-fMRI data in terms of image quality (Fig. 2) and improvement to map RSNs (Fig. 3andFig. S3). The results show that acceptable spatial blur is induced to the data whereas tSNR is boosted much more. Note that patch processing with a small image window (3 × 3) was used in the processing, leading to a 2D Casorati matrix with a size of 9 × 310. The large matrix size along time, according to the RMT, leads to a narrow Tracy-Widom distribution that lowers the uncertainty of the largest noise singular value and ensures accurate noise estimation and suppression. Nevertheless, removing the noise components from data is not the final deal because the signal component is also contaminated by noise and deviated from its true value. Therefore, further cleaning the signal components is another important step in our RMT-PCA, which distinguishes our method from others. A more detailed discussion on the advantages of our RMT-PCA approach and comparison with other PCA methods including NORDIC can be found in our publication (Zhu et al., 2022). Finally, although our RMT-PCA was applied to data obtained from a single-loop radiofrequency (RF) surface coil, it does not limit its application to data from array coils (after g-factor correction) or volume coils.

Motion is a major source of variation in fMRI. In this work, we explored the commonly used SPM rigid motion estimation as well as the novel non-rigid motion estimation strategy. As the performance of SPM 3D rigid motion estimation algorithm deteriorates when movement is small (Friston et al., 1995) and small distortion may happen, a non-rigid algorithm is complementary. The non-rigid motion estimation algorithm adopted in this work is based on patch registration using phase correlation in Fourier transformed domain against the reference template. The non-linear sub-pixel shift map is derived from the rigid shift information from each patch (Fig. S7). We found the combination of rigid and non-rigid motion registration led to the most accurate result (Fig. 2H-2J). Further examination of our fMRI data also shows a translation along the phase encoding dimension and this translation fluctuation varies among slices (Fig. 2K). Therefore, a 2D translation correction was implemented on our data. However, this phase fluctuation may be machine specific (Varian) and the 2D correction may not be necessary to data acquired from other vendors.

Additionally, preprocessing of anatomical image and reference image (brain atlas images in this study) with fMRI images requires extra care if group-level analysis is needed. This is particularly true for the mouse with a very small brain size and thin cortical layers. The goal of this step is to align the functional image to the group averaged reference image or brain atlas through the high-resolution and texture-rich anatomical image. Our contribution in this work is the construction of an optimized pipeline that yields high-quality alignment among three kinds of data (Fig. 2PandFig. S2). First, the transverse magnetic field (B_1_) produced by an RF coil bias correction and non-brain tissue removal (not required to be perfect) on anatomical images are critical for the following image co-registrations. Second, the key idea for a successful image co-registration is registering the texture-rich image to a less rich image but with similar contrast, such as anatomical image to functional image, and atlas to anatomical image. Rigid transformation is applied on anatomical to functional registration by assuming a small or correctable EPI distortion. For EPI images with large distortion, nonlinear registration may be useful to unwrap EPI images to anatomical images (Wang et al., 2017) but is yet to be tested on small animals. Meanwhile, non-linear registration using symmetric normalization (SyN) (Avants et al., 2008) following linear registration (rigid and affine) is conducted on atlas to anatomical registration, yielding to high fidelity alignment. Forward and inverse transformation chains can be constructed to further align atlas to individual EPI (good for ROI-based connectivity analysis) or individual EPIs to one atlas (good for seed-based connectivity analysis), respectively.

### 4.3 Connectivity analysis

Atlas-based connectivity analysis (Maldjian et al., 2003) was used in this study by exploiting the 3D reference atlas provided in the Allen mouse brain common coordinate framework (Dong, 2008;Q. X. Wang et al., 2020). The standardized anatomical parcellation in Allen mouse atlas facilitates brain-wide network analysis in relation to the other facets of neuroarchitecture (Leergaard & Bjaalie, 2022), such as cell-class-specific connections (Harris et al., 2019), adeno-associated virus (AAV) based axonal projections (Oh et al., 2014), and gene expression based connectome (Lein et al., 2007). In this work, robust RSNs of mouse brain from large brain areas to thin cortical layers indicate the hierarchical organizations with varied brain functions (Figs. 3-5). The distinct patterns in RSNs across cortical layers evidence the non-uniform spontaneous neuronal activities underlay, paving the way to study brain-wide neural circuits and synchronization non-invasively at the mesoscopic scale. For example, neuronal signals from the thalamus are projected mainly to layer 4 of the cortex, whose outputs are relayed to layer 2/3 for further information processing. Layer 2/3 then projects signal to layer 5 from where signals go to layer 6 and subcortical targets (Jabaudon, 2017). Laminar RSNs as shown inFigure 3(right panels) generally capture such undirected layer-specific circuit connections with high fidelity and have a potential to unveil directed signal flow in mesoscopic neural circuits by incorporating Granger causality analysis (Roebroeck et al., 2005;Seth et al., 2015;X. Wang et al., 2020).

Instead of seed-based connectivity analysis as performed in this work, ROI-based analysis is suitable for simultaneous brain-wide network investigation. Correlations (links) between all pairs of predefined ROIs (nodes) form the adjacency matrix (Fig. 6), which can be used to calculate graph measures, such as degree and similarity, clustering and modularity, functional hubs, etc. (Rubinov & Sporns, 2010). Undoubtedly, graph measures at cortical layer and nucleus level can provide new insights in understanding the functional topological organization and neuro-computational units in the brain.

### 4.4 Neural connectivity between cortical layer and white matter

In this study, we observed neural connections between the cortical regions and bundled white matter fibers based on the high-resolution rs-fMRI and advanced signal processing approach (seeFigs. 3and5). The results reinforce that white matter functional connectivity provides rich spatiotemporal information, which is an important part of the functional brain organization (Gore et al., 2019;P. Wang et al., 2022). More interestingly, the white matter connections shown in this work are layer specific, with strong fiber connection to superficial layers in the RSN of the lower limb (Fig. 3), and to middle layers for the RSN based on the SSp-upper limb seeding which agrees with the*ex vivo*results based on the AAV viral tracer projection (Fig. 7C). Such connections could be very challenging to detect using traditional rs-fMRI and analysis approaches based on ROI seeding owing to low spatial resolution (i.e., partial volume effect), the lack of layer specificity, and inaccurate image co-registration between subjects. In addition, the detailed comparison between corpus callosum RSNs with composite AAV viral projection connectome covering multiple SS areas indicates the complex functional fluctuations in this connectivity highway due to the busy communication among local and remote brain areas (Fig. 7C). The discrepancy between functional and anatomical connectivity is also observed in other studies (Wang et al., 2021). Nevertheless, the robustness and accuracy of using the high-resolution and layer-specific rs-fMRI for mapping the functional connectivity converging both cortical and bundled fibers require thorough investigation and evaluation (Gawryluk et al., 2014;Gore et al., 2019).

### 4.5 Relation with neural synchronization

Rs-fMRI based on BOLD contrast measures the hemodynamic responses and is assumed to reflect a low-pass filtering of the higher frequency neural activity through the complex neurovascular coupling (Honey et al., 2009). However, a recent study showed that spontaneous BOLD signals are specifically attributable to an infra-slow brain process (<0.1 Hz) that could travel through specific cortical layers with unique cross-laminar temporal dynamics and are distinct from high-frequency local field potentials (Mitra et al., 2018). This evidence further supports that spontaneous BOLD serve as an excellent surrogate of intrinsic neural synchronization even at laminar specificity, consistent with the laminar RSNs observed in this study. Nevertheless, it is also shown that the infra-slow brain process (<0.1 Hz) is state-dependent (Mitra et al., 2018), with signal directionality shifts in distinct cross-layer relation across wake versus anesthesia (Marder, 2012). Therefore, the laminar RSNs obtained under dexmedetomidine sedation in this study may not be the same as the laminar connection patterns in awake state. The differences of laminar RSNs between two states are worth future investigations. Another study also reveals intrinsic frequency clusters in the rs-fMRI signal within the 0.01–0.25 Hz range, with frequency peaks at 0.028 Hz and 0.08 Hz associated with physiological fluctuations and metabolic processes, respectively (Yuen et al., 2019). It would be interesting to study whether these frequency clusters present at laminar level. Further, it is also critical to investigate the information flow from layer to layer, and their overall influence on the local brain region as a whole. For example, it is known that in mouse brain, signals from the sub-cortex are relayed to layer 5, where pyramidal neurons further project the signals to layer 2/3 and layer 4, and after processing, the signals are transferred back to layer 5 and 6. To differentiate this time-dependent information, more advanced analysis such as causality analysis is needed. The evolution of RSNs across time is another topic that needs exploration. As the neural activity is not stationary within seconds to minutes (Cabral et al., 2023;Chang & Glover, 2010), the functional connectivity pattern may also change at a different time window.

Another interesting question related to BOLD mechanism is its state dependence. In resting state, a close relation between rs-fMRI BOLD and infra-slow neural synchronization seems to be true, leading to layer-specific RSNs even with GE-EPI sequence that is known to suffer from large draining vein effect in task fMRI. The mitigation of draining vein effect in resting state may be due to two factors. First, compared to the use of tasks or stimulation, resting state is less demanding of global oxygen supply and compensation, inducing localized BOLD changes. Second, the large vessel contamination in the evoked BOLD responses might be filtered out by the temporal correlation analysis owing to either much weak temporal synchronization or too slow temporal fluctuation in the large vessels. Therefore, the T_2_^*^BOLD specificity may not be a hurdle for laminar-level analysis. In contrast, the hemodynamic changes at working state seem to be less specific and imaging sequence dependent. This may be due to the dramatically increased metabolic demand of oxygen and cerebral blood flow (perfusion) that trigger extra physiological changes. To understand the BOLD mechanism and its difference between resting and working states, other techniques such as optical imaging that could provide neuronal and vascular information should be combined with rs-fMRI to gain insights.

## 5 Conclusion

Efforts to investigate spontaneous BOLD signal dynamics using high-resolution resting-state fMRI (rs-fMRI), and thus the neural synchrony and connectivity, are essential to our understanding of neural circuits at cortical layer and sub-cortical nucleus level. In this work, we demonstrated the feasibility of achieving brain-wide laminar-specific functional connectomes in a mouse model by applying an advanced data processing pipeline to high-resolution mouse rs-fMRI with enhanced temporal signal-to-noise ratio, minimized spatial blur, and augmented subject-level alignment. The distinct functional connectivity patterns across cortical layers evidence the non-uniform spontaneous neuronal activities underlay, paving the way to study brain-wide neural circuits and synchronization from macroscopic to mesoscopic scale. It is critical to further push the rs-fMRI resolution for improving the specificity and fidelity of mapping laminar-specific connectomes. Finally, the advanced data processing pipeline can be applied to process task-evoked fMRI or adapt to human brain fMRI applications.

## Supplementary Material

Supplementary Material

## Data Availability

MRI data are available from the corresponding author upon request and the computer code is available onhttps://github.com/zw1989.
