# Supplementary material for: A robust approach for analyzing and mapping hierarchical brain connectome towards laminar-specific neural networks
Source: Imaging Neurosci (Camb). 2025 Apr 22;3:imag_a_00543. doi: 10.1162/imag_a_00543 (PMC12320003; doi:10.1162/imag_a_00543)
Supplement: Supplementary Material [file imag_a_00543-supp.pdf]

Supporting Information (SI) for

A robust approach for analyzing and mapping hierarchical brain connectome towards laminar-specific neural networks

Wei Zhu, Guangle Zhang, Xiao-Hong Zhu, Wei Chen*
Center for Magnetic Resonance Research, Department of Radiology, University of Minnesota, Minneapolis, Minnesota, USA.

**Corresponding Author: Wei Chen, PhD.
Email: chenx075@umn.edu

This PDF file includes:

Supporting text
Figures S1 to S7
SI References

Supporting Information Text

Materials and Methods

Animals and MRI scan conditions

Thirteen wild type mice (C57BL/6, 7 male/6 female) were scanned under a protocol approved by the University of Minnesota IACUC. All mice were inducted with 4% isoflurane mixed in O_2:N_2O (30%:70%) gas and anesthetized with 1.5% isoflurane during the preparation. Bite bar and ear bars were positioned carefully to fix the mouse head without pain. Lidocaine gel was smeared on the ear bars to release possible stress and pain during the setup. A respiration (pressure) pad was placed under the mouse chest to monitor the respiration rate. A water bath was surrounded the mouse to keep the body temperature at a range of 36.6-38.0°C measured by a rectal thermometer. The animals' physiology was monitored and recorded using a MR-compatible monitoring and gating system for small animals (Model 1030, SAII) throughout the study. After a mouse was transferred into the MRI magnet, a bolus of dexmedetomidine (0.3 mg/kg) was administered intraperitoneally. Meanwhile, isoflurane dose was reduced at a rate of 0.15% per two minutes. The anesthesia was completely switched to dexmedetomidine from isoflurane in 20 mins after bolus injection and then followed by 0.6 mg/kg/hr infusion (Chu et al., 2021) during the rs-fMRI study. The rs-fMRI data acquisition started once the mouse respiration rate stabilized at > 140 BPM.

MRI experiments

The MRI experiments were conducted on a 9.4T/31cm animal scanner (Varian/VNMRJ) using a single loop (1.5 cm diameter) transceiver RF surface coil. Single shot 2D gradient-echo echo planar image (GE-EPI) based rs-fMRI data were obtained with repetition time (TR) = 1000 ms, echo time (TE) = 16 ms, matrix size = 96 × 48, kzero = 22 (echo signal that will be assigned as the center of the k-space), field of view (FOV) = 2.4 × 1.2 cm^2, nominal RF pulse flip angle (FA) is the Ernst angle = 55°, in-plane resolution = 0.25 x 0.25 mm^2, slice thickness = 0.5 mm, bandwidth = 5208 Hz/pixel, and echo spacing = 0.568 ms. a total of 12 slices in axial orientation were acquired for 5 mice without field map data and 16 slices were acquired for 8 mice. Two to five rs-fMRI runs (5.2 mins per run) were repeated for each subject, each comprising 310 GE-EPI volumes and leading to a total fMRI acquisition time from 10.3 mins to 25.8 mins.

Field map data were acquired for mice with 16 GE-EPI slices (for 8 mice) based on the dual echo-based GRE imaging pulse sequence with TR = 120 ms, TE_1/TE_2 = 3 ms/3.5 ms, FA = 43°, bandwidth = 1042 Hz/pixel. Matrix size and FOV were the same as those in EPI. T_2 weighted anatomical images were acquired with TR = 5000 ms, effective TE= 30 ms, matrix = 256 × 128, echo train length = 8, average number = 5, bandwidth = 781 Hz/pixel, FA = 100°/200° with the same brain coverage.

Rs-fMRI data preprocessing

The rs-fMRI data were preprocessed with the proposed fMRI pipeline shown in **Fig. 1**. Four preprocessing branches for field map data, functional data, anatomical data, and atlas data were included, and only the functional data preprocessing branch was necessary and the other three were optional. The main features of the functional data processing branch were 1) applying random matrix theory-based principle component analysis (RMT-PCA) denoising with patch processing (patch size 3 × 3 × 1 was used) to effectively remove thermal noise without inducing severe spatial blur (Zhu et al., 2022), 2)

combining rigid an non-rigid registration steps to enhance motion estimation accuracy, and 3) minimizing the spatial interpolation steps by combining voxel shifts from various sources such as B_0 field distortion, motion, resampling, etc. into one step to minimize image blur. In addition, rigid motion with six degrees of freedom (3 translations and 3 rotations) was estimated using the realign function in SPM (Ashburner, 2012) with optimized parameters for mouse, i.e., estimation quality = 1, spatial smoothing size (FWHM) = min(epi resolution) or 0, sampling distance (sep) = max(FOV)/50 as a good thumb of rule, the degree of the B-spline interpolation (interp) = 7. Non-rigid motion estimation was performed based on pixel shift in small patches derived from phase-correlation between normalized Fourier spectra of the patch images. The algorithm was rewritten based on an existing function (Pachitariu et al., 2016) with improved performance on MRI images. The patch size used in this work was 32 x 32 x 1 and hop size of the patch was 4 x 4 x 1 (**Fig. S7**). As non-rigid registration follows rigid registration, the maximum pixel shift was set to 3 in both dimensions. One special situation in this work was the estimation of pixel shift along phase encoding dimension for each slice on each EPI run. The fluctuation along phase may be scanner specific and this operation is optional. A binary brain mask indicating which voxels to be used in motion estimation was generated by thresholding the average EPI images along time. The background threshold was determined at an intensity of $I_t = I_2 + \mu(I_{98} - I_2)$, where I_2 and I_{98} were the 2nd and 98th percentile of the average EPI, respectively, and μ was a tuning parameter taking 0.2 by default as a good rule of thumb (Jenkinson, 2003). The quality of rs-fMRI data was then controlled with four methods: 1) the overlay image of the first and last volume of the EPI images, 2) tSNR map, 3) voxel plot with the horizontal axis the volume number and the vertical axis the brain voxels (Power, 2017), and 4) framewise displacement based on motion parameters (Power et al., 2012). The framewise displacement was also useful to detect outliers with the adjusted Carling's box-plot rule (Carling, 2000). The identified outlier volumes were directly removed from the rs-fMRI time series. If more than 20% of the volumes were identified as outliers in a time series, this time series was discarded. As the animals were anesthetized, head motion was limited and the outliers took less than 1% of the EPI run. As upsampling has been proved to be useful in preserving fine-scale details that are present in the raw rs-fMRI data (Allen et al., 2022; Kang et al., 2007; Kay et al., 2019), we upsampled the data to the target resolution of [0.1 mm, 0.1 mm, 0.5 mm]. We also filtered the undistorted and slice time corrected rs-fMRI data with frequency ≤ 0.01Hz, accounting for residual motion and low frequency signal drift.

Field map data, if provided, should include magnitude image and at least two phase images with different echo times. The phase images were first unwrapped by using the prelude function of FSL (Jenkinson, 2003) and the difference between the unwrapped phase images was the B_0 map. The B_0 map was further smoothed before being used to correct EPI distortions with other voxel shift sources.

The main feature of the anatomical data preprocessing branch for the subject-level alignment was the flexible and robust registration strategy built on Advanced Normalization Tools (ANTs, (Avants et al., 2014). The image co-registration consisted of two-folds incorporating the averaged undistorted functional images, the anatomical images (T_1 or T_2 weighted), and reference image or brain atlas: 1) co-registering anatomical images to average EPI images with only rigid transformation (anat2func), and 2) co-registering reference image or atlas to anatomical images with deformable transformation (atlas2anat) based on symmetric normalization (SyN) (Avants et al., 2008; Avants et al., 2014). Two transformations were combined and applied to the atlas template and annotation images to map them to the EPI space. Inversely, the preprocessed EPI

images could also be aligned with atlas images. To ensure the co-registration quality, especially for the non-linear transformations, anatomical data were first corrected for intensity non-uniformity due to the biased RF magnetic field (B_1) (Tustison et al., 2010). Then the corrected data were denoised using non-local means if SNR was low and enhanced in terms of sharpness and contrast (image enhancement was also performed on functional and atlas images). Then a brain mask was generated by using the same mask generation function as used in functional data preprocessing. Non-brain tissues were stripped from the B_1 corrected, denoised, and image enhanced images using the AFNI 3dSkullStrip function (Cox, 1996; Cox & Hyde, 1997).

Taking advantage of the Allen mouse atlas (Lein et al., 2007), we performed atlas-based seed connectivity analysis by seeding brain regions at various hierarchical levels (down to cortical layers and thalamic nucleus) on the preprocessed rs-fMRI data to generate hierarchical RSNs. Fisher z-transformation of the Pearson correlation coefficient was calculated to display the resting state networks.

Supporting Information Figures

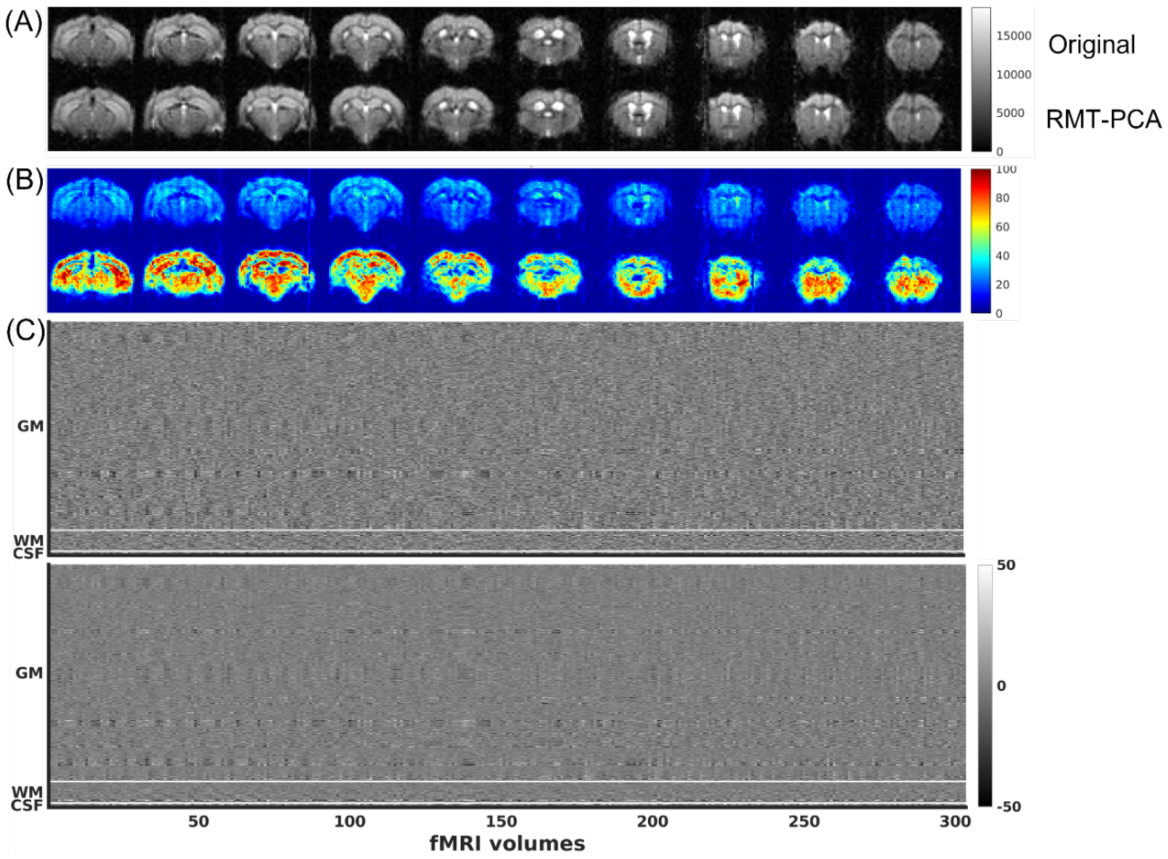

Figure S1. Comparing performance of with (low rows) and without (top rows) RMT-PCA denoising on one EPI run for a randomly selected mouse in terms of (**A**) image quality, (B) temporal signal-to-noise ratio (tSNR) map, and (**C**) voxel plot (Power, 2017). The top row in each panel is the result from raw rs-fMRI data as reference. Dramatic thermal noise was removed from the spatial-temporal rs-fMRI data (**C**), enhancing the tSNR by about 2.2 times (**B**) without introducing obvious spatial blur (**A**). Note that the voxel plots are arranged based on grey matter (GM), white matter (WM), and cerebrospinal fluid (CSF).

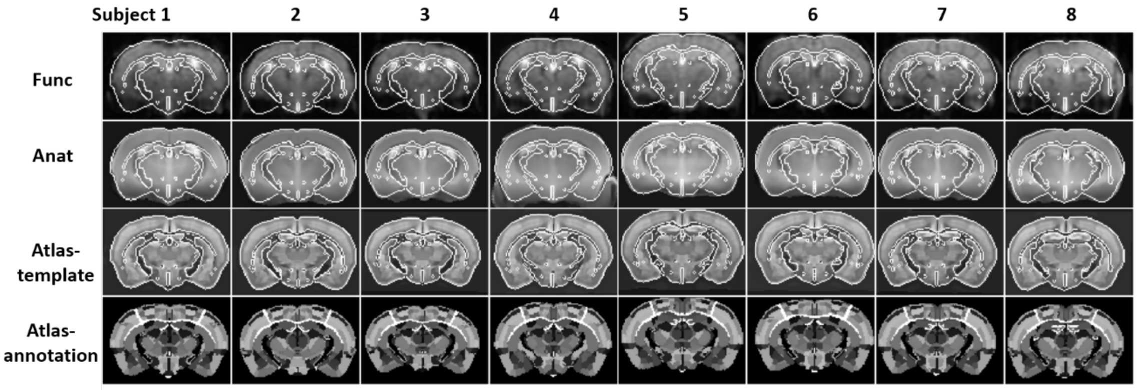

Figure S2. Co-registered Allen mouse brain atlas and anatomical images (Anat) to average functional EPI images (Func) for eight randomly selected mice. One brain slice is used for demonstration. Rigid transformation was used to register Anat to Func. Non-linear transformation was used to register atlas to Anat and then to Func. Inverse transformation can also be performed to map Func to the original atlas space, i.e., subject normalization. Outlined mouse brain atlas is overlayed on Func and Anat for comparison.

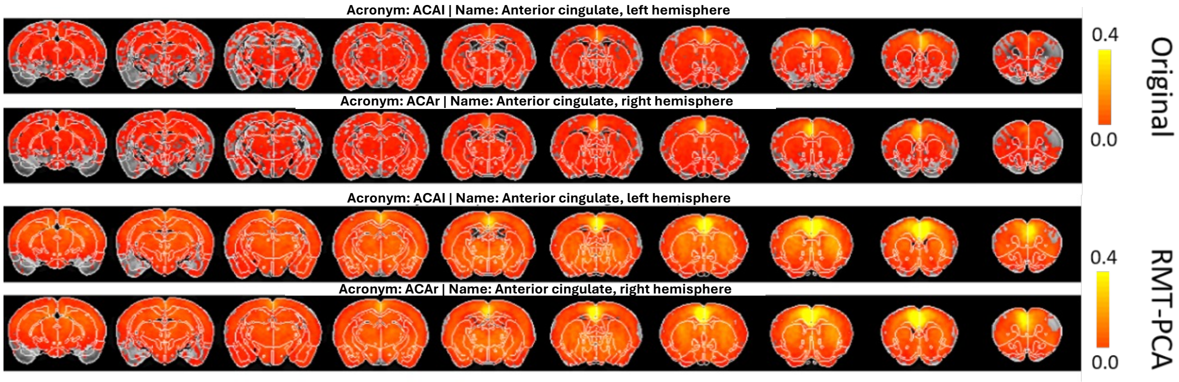

Figure S3. An example of resting state networks with seeding at anterior cingulate area (ACA) in the left and right hemispheres obtained from (**A**) original EPI data, and (**B**) RMT-PCA denoised EPI data as shown in **Fig. 3**, but without correlation threshold.

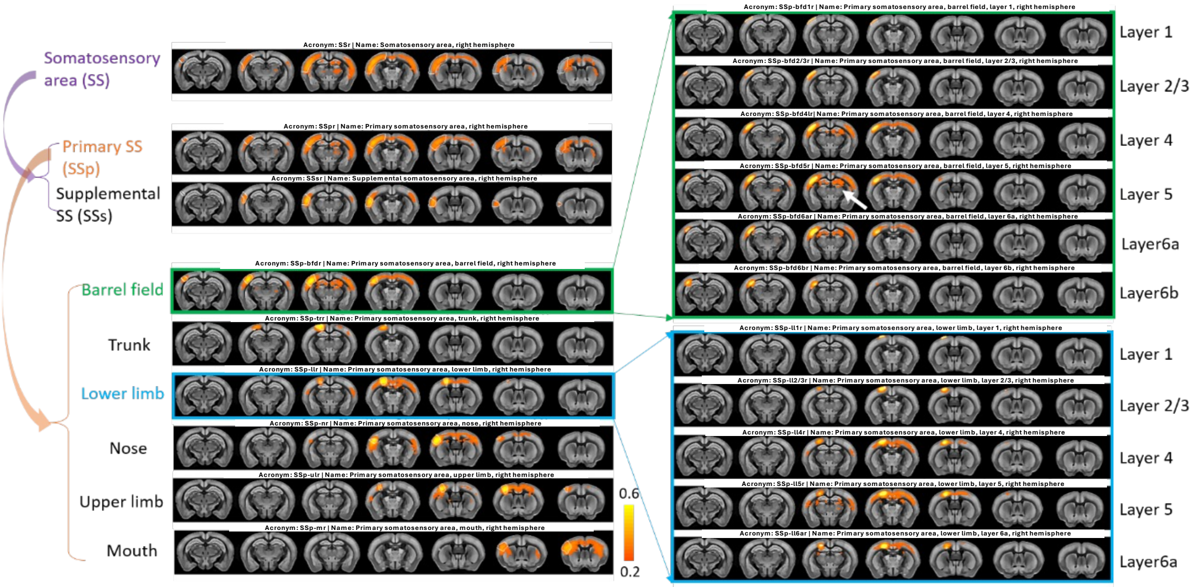

Figure S4. Hierarchical RSNs with seeding in somatosensory (SS) area in right hemisphere at different structure levels based on the RMT-PCA denoised data.

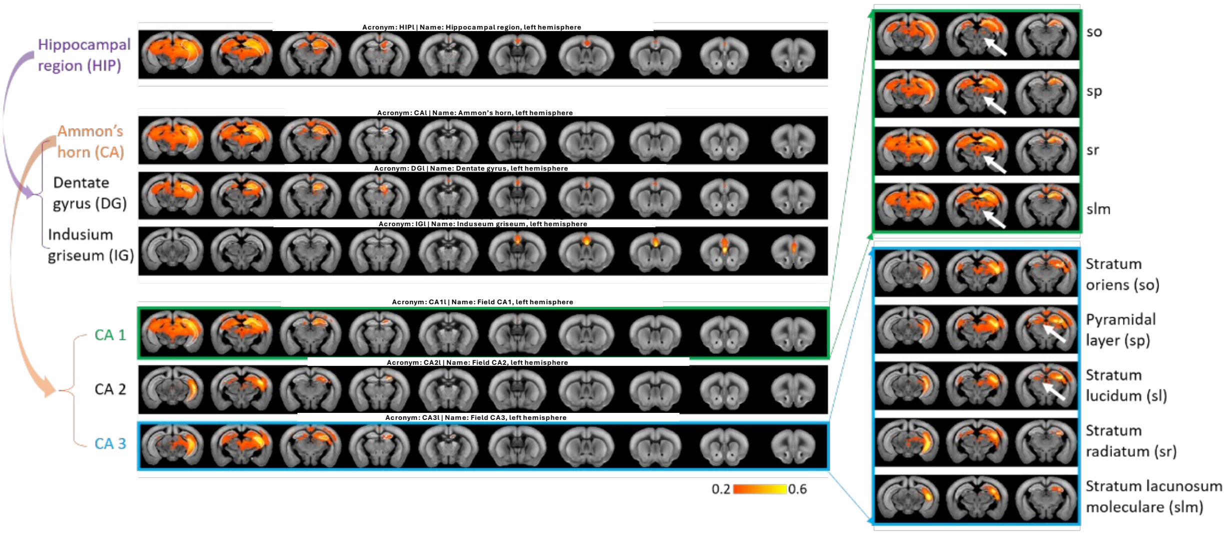

Figure S5. Hierarchical RSNs with seeding in hippocampal region (HIP) at different structure levels in left hemisphere. RSNs with seeding at the whole HIP share a large amount of similarity with those seeding at Ammon's horn, and then seeding at CA 1 that accounts for most of the HIP area. Seeding at different layers of CA 1 and CA 3 reveals identifiable connectivity patterns. Note that due to the main connections when seeding at CA 1-3 cover a limited brain area, only the first three slices are displayed to show the comparison on the right panels. The seeding areas are outlined by white dashed lines.

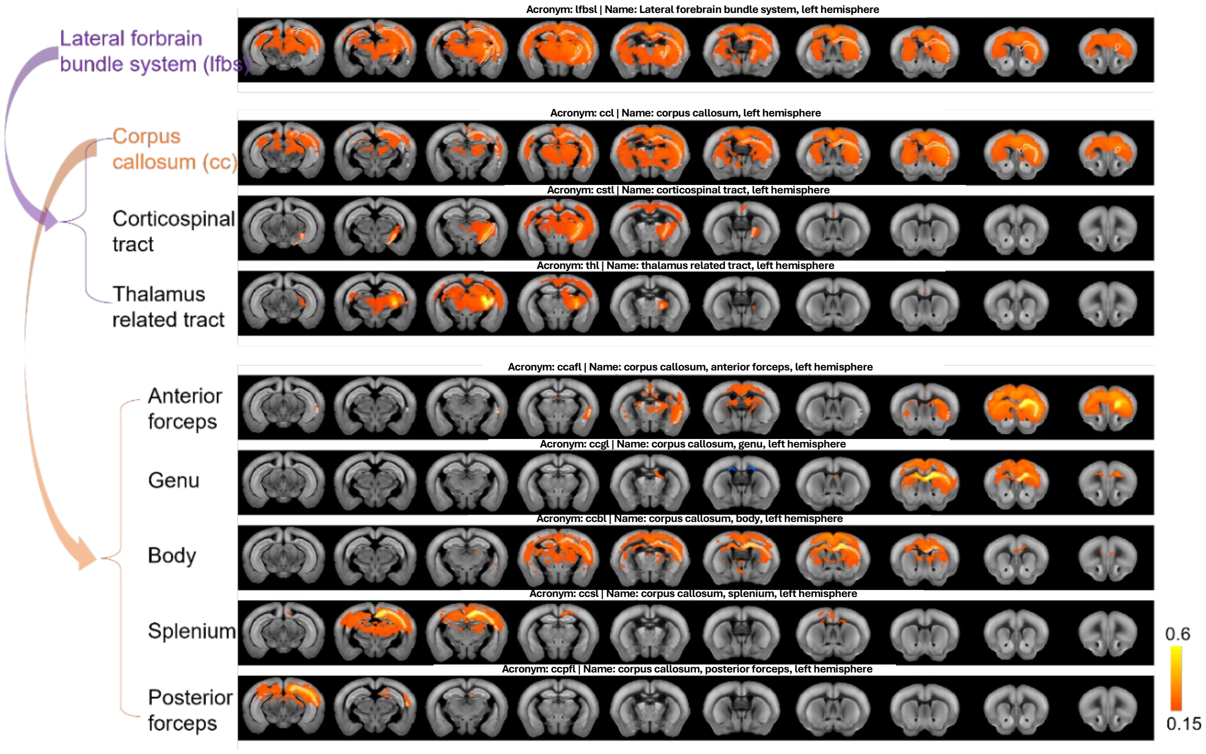

Figure S6. Hierarchical RSNs with seeding in lateral forebrain bundle system (lfbs, white matter) at different structure levels in left hemisphere. Whole brain connections when lfbs is the seed. Localized and stronger connectivity appears when sub-white matter bundles are seeded. The seeding areas are outlined by white dashed lines.

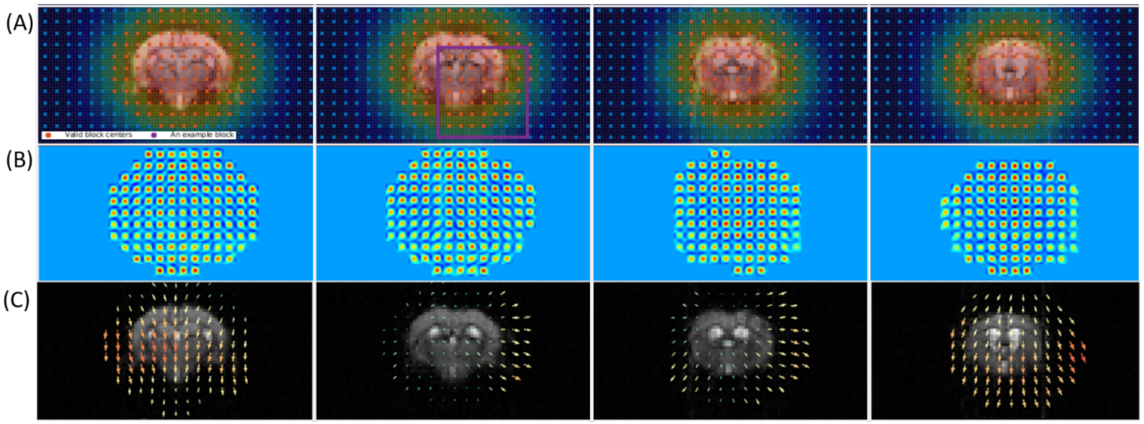

Figure S7. Intermedium outputs from non-rigid motion estimation algorithm. (**A**) Example patch/block size (purple square) and the valid patch/block distribution (orange dots). (**B**) Phase correlation map for each patch. (**C**) Pixel shift map estimated based on the phase correlation map.

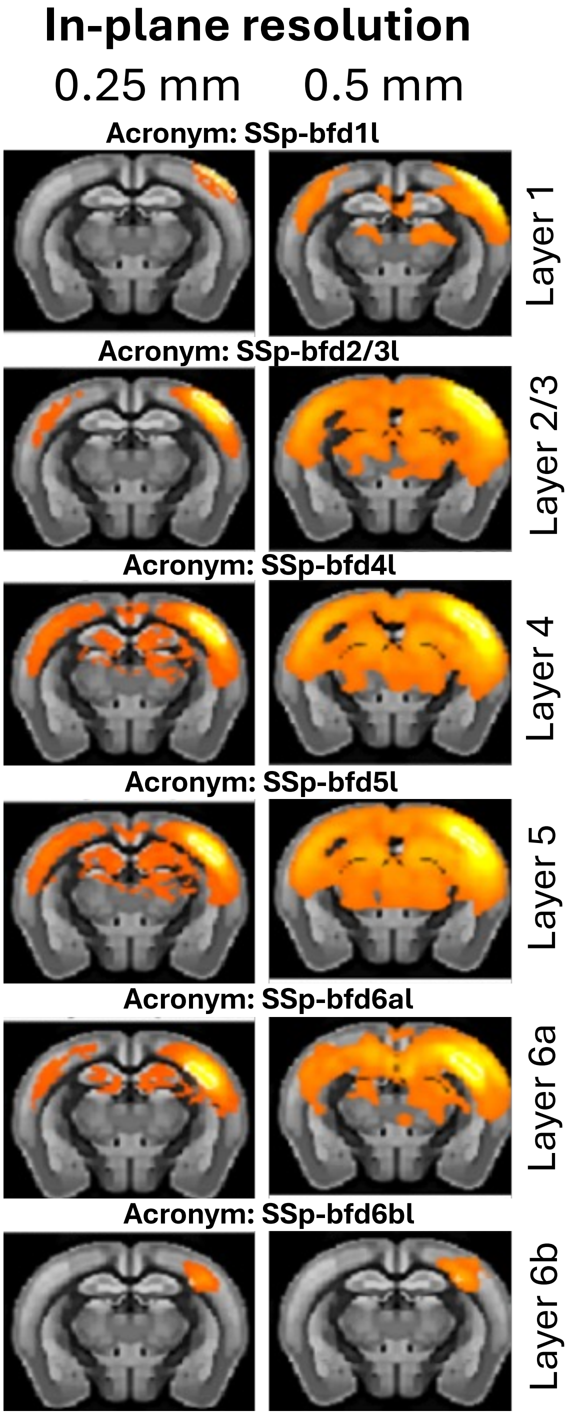

Figure S8. Specification of the RSNs for high in-plane resolution of 0.25 mm and low in-plane resolution of 0.5 mm. RSNs from low resolution data are less specific.

SI References:

Allen, E. J., St-Yves, G., Wu, Y. H., Breedlove, J. L., Prince, J. S., Dowdle, L. T., Nau, M., Caron, B., Pestilli, F., Charest, I., Hutchinson, J. B., Naselaris, T., & Kay, K. (2022). A massive 7T fMRI dataset to bridge cognitive neuroscience and artificial intelligence. *Nature Neuroscience*, *25*(1), 116-+. https://doi.org/10.1038/s41593-021-00962-x

Ashburner, J. (2012). SPM: a history. *Neuroimage*, *62*(2), 791-800. https://doi.org/10.1016/j.neuroimage.2011.10.025

Avants, B. B., Epstein, C. L., Grossman, M., & Gee, J. C. (2008). Symmetric diffeomorphic image registration with cross-correlation: evaluating automated labeling of elderly and neurodegenerative brain. *Med Image Anal*, *12*(1), 26-41. https://doi.org/10.1016/j.media.2007.06.004

Avants, B. B., Tustison, N. J., Stauffer, M., Song, G., Wu, B. H., & Gee, J. C. (2014). The Insight ToolKit image registration framework. *Frontiers in Neuroinformatics*, *8*. https://doi.org/ARTN 44
10.3389/fninf.2014.00044

Carling, K. (2000). Resistant outlier rules and the non-Gaussian case. *Computational Statistics & Data Analysis*, *33*(3), 249-258. https://doi.org/Doi 10.1016/S0167-9473(99)00057-2

Chu, Q., Zhu, K., Bai, Y., Shang, H., Zhang, D., Zhao, M., Zheng, P., & Jin, X. (2021). A Single Low Dose of Dexmedetomidine Efficiently Attenuates Esketamine-Induced Overactive Behaviors and Neuronal Hyperactivities in Mice. *Front Hum Neurosci*, *15*, 735569. https://doi.org/10.3389/fnhum.2021.735569

Cox, R. W. (1996). AFNI: software for analysis and visualization of functional magnetic resonance neuroimages. *Comput Biomed Res*, *29*(3), 162-173. https://doi.org/10.1006/cbmr.1996.0014

Cox, R. W., & Hyde, J. S. (1997). Software tools for analysis and visualization of fMRI data. *NMR Biomed*, *10*(4-5), 171-178. https://doi.org/10.1002/(sici)1099-1492(199706/08)10:4/5<171::aid-nbm453>3.0.co;2-l

Jenkinson, M. (2003). Fast, automated, N-dimensional phase-unwrapping algorithm. *Magn Reson Med*, *49*(1), 193-197. https://doi.org/10.1002/mrm.10354

Kang, X. J., Yund, E. W., Herron, T. J., & Woods, D. L. (2007). Improving the resolution of functional brain imaging: analyzing functional data in anatomical space. *Magnetic Resonance Imaging*, *25*(7), 1070-1078. https://doi.org/10.1016/j.mri.2006.12.005

Kay, K., Jamison, K. W., Vizioli, L., Zhang, R., Margalit, E., & Ugurbil, K. (2019). A critical assessment of data quality and venous effects in sub-millimeter fMRI. *Neuroimage*, *189*, 847-869. https://doi.org/10.1016/j.neuroimage.2019.02.006

Lein, E. S., Hawrylycz, M. J., Ao, N., Ayres, M., Bensinger, A., Bernard, A., Boe, A. F., Boguski, M. S., Brockway, K. S., Byrnes, E. J., Chen, L., Chen, L., Chen, T. M., Chin, M. C., Chong, J., Crook, B. E., Czaplinska, A., Dang, C. N., Datta, S., Dee, N. R.*, et al.* (2007). Genome-wide atlas of gene expression in the adult mouse brain. *Nature*, *445*(7124), 168-176. https://doi.org/10.1038/nature05453

Pachitariu, M., Stringer, C., Dipoppa, M., Schröder, S., Rossi, F., Dalgleish, H., Carandini, M., & Harris, K. (2016). Suite2p: beyond 10,000 neurons with standard two-photon microscopy. *bioRxiv*. https://doi.org/10.1101/061507

Power, J. D. (2017). A simple but useful way to assess fMRI scan qualities. *Neuroimage*, *154*, 150-158. https://doi.org/10.1016/j.neuroimage.2016.08.009

Power, J. D., Barnes, K. A., Snyder, A. Z., Schlaggar, B. L., & Petersen, S. E. (2012). Spurious but systematic correlations in functional connectivity MRI networks arise from subject motion. *Neuroimage*, *59*(3), 2142-2154. https://doi.org/10.1016/j.neuroimage.2011.10.018

Tustison, N. J., Avants, B. B., Cook, P. A., Zheng, Y., Egan, A., Yushkevich, P. A., & Gee, J. C. (2010). N4ITK: improved N3 bias correction. *IEEE Trans Med Imaging*, *29*(6), 1310-1320. https://doi.org/10.1109/TMI.2010.2046908

Zhu, W., Ma, X., Zhu, X. H., Ugurbil, K., Chen, W., & Wu, X. (2022). Denoise Functional Magnetic Resonance Imaging with Random Matrix Theory Based Principal Component Analysis. *IEEE Trans Biomed Eng*, *PP*. https://doi.org/10.1109/TBME.2022.3168592
